# Predicting Migratory Survival in a Songbird Hybrid Zone Using Machine Learning

**DOI:** 10.21203/rs.3.rs-8108392/v1

**Published:** 2025-12-03

**Authors:** Sarah A. Vastani, Stephanie A. Blain, Hannah Justen, Wendy E. Easton, Kira E. Delmore

**Affiliations:** Texas A&M University; Columbia University; University of California, Davis; Environment and Climate Change Canada; Columbia University

## Abstract

Extrinsic postzygotic isolation—selection against hybrids between populations that have undergone divergent ecological adaptation—is hypothesized to play a central role in speciation but is notoriously difficult to demonstrate, as it requires evidence that ecologically relevant traits influence hybrid fitness. We addressed this challenge using individual radio tracking and machine learning in a hybrid zone between two songbirds where differences in seasonal migration are thought to serve as extrinsic isolating barriers. Using detection rates as a proxy for survival, we built a random forest classification model to predict survival from a set of genetic, morphological, and behavioural traits. The model achieved moderately high accuracy (72%), with better prediction for birds that did not survive migration. Alongside body condition and study year, the traits that contributed most to classification were genetic ancestry, genetic heterozygosity, and fall orientation. These traits had non-linear effects on survival, with some values actually predicting higher survival in hybrids. Together, these results suggest that a bird’s hybrid class—defined by ancestry and heterozygosity—and its initial migratory direction affect migratory survival. Interactions with morphology are important and along with non-linear associations between traits and survival, reflect the complexity of relationships between behavioral traits and fitness. Our model not only provides a complete picture for the role ecological selection on migration plays in speciation, but also supports growing evidence that some hybrids may benefit from admixture.

## Introduction

Ecological speciation is the process through which divergent adaptation contributes to the evolution of reproductive isolation between two or more species [1, 2]. This form of reproductive isolation can evolve via the accumulation of extrinsic postzygotic barriers, which are expressed when hybrid fitness is reduced in nature [3]. A key requirement for demonstrating extrinsic postzygotic isolation is connecting ecologically relevant traits that are under divergent selection to reductions in hybrid fitness. This requirement can be challenging to meet, as estimating fitness in natural populations is notoriously difficult [4]. Even if fitness can be quantified, it can be difficult to connect specific traits to reductions in fitness because incipient species have often diverged in many traits, generating correlations between them [5–7]. Hybrid zones, regions where incipient species come into contact and hybridize, can help to address this [8, 9]. Hybridization and subsequent recombination can generate a broad range of trait values as well as highly variable combinations of traits, which offers an opportunity to observe how these trait combinations perform in nature [10].

Although hybridization offers an opportunity to observe and connect patterns of trait and fitness variation, the nature of relationships between traits and fitness can still make them challenging to characterize. Fitness landscapes can be complex, with multiple local maxima [11, 12]. Generally, the parental species’ phenotypes are expected to have higher fitness than hybrids, but this may not be true in hybrid zone environments where there may be novel trait combinations or intermediate phenotypes that perform well [13–15]. Furthermore, traits may have interactive effects on fitness, so that the effect of one trait on fitness depends on others [9]. Therefore, it can be difficult to disentangle which traits are important for fitness variation in hybrid zones, particularly if interactive effects are non-linear or if several traits are involved.

In the present study, we used a hybrid zone between two subspecies of Swainson’s thrushes, combined with a modelling approach based in machine learning (ML), to overcome challenges associated with identifying traits that contribute to ecological speciation. The two Swainson’s thrush subspecies form a migratory divide; the inland subspecies migrates via eastern North America to South America, while the coastal subspecies migrates to Mexico and Central America along the Pacific coast [16]. Seasonal migration has long been hypothesized to act as an extrinsic, post-zygotic barrier in migratory divides, with hybrids exhibiting intermediate or mismatched migratory traits compared to parental forms, reducing their fitness [17, 18]. Work on the Swainson’s thrush arguably provides the most direct test of this hypothesis, with parental forms taking different routes on migration [16], hybrids exhibiting more variability in their migratory routes including intermediate and mismatched traits [19, 20], and mark recapture models showing hybrids and coastal backcrosses survive migration at lower rates than the parental subspecies [21]. Nevertheless, attempts to identify specific migratory traits that reduce hybrid fitness have thus far been unsuccessful. Specifically, the subspecies have diverged in a suite of traits that are associated with long-distance migration, ranging from behaviours, such as migratory timing, to morphological traits, including wing shape [16, 22]. Hybrids exhibit high variability and diverse combinations of migratory traits [19, 20]. However, these traits do not show linear or quadratic relationships to survival in hybrids, suggesting that if there are links between traits and fitness, they are complex or interactive [21].

ML approaches can be powerful for analyzing high-dimensional ecological data and revealing complex, non-linear and interactive relationships that traditional statistical methods fail to detect [23]. Random Forest is one example. This approach can be used to classify an outcome based on a multidimensional set of features, and involves constructing multiple decision trees during training and combining their predictions to improve accuracy and reduce overfitting [24] (Fig. S1). We used a Random Forest ML model to examine the relationships between survival and a suite of morphological and behavioural traits in Swainson’s thrush hybrids. We used patterns of individual detections from an automated radio telemetry network as a proxy for survival and to estimate individual behaviours on fall migration [25, 26]. By leveraging these technological and computational approaches, we aim to identify key traits and interactions that shape survival on migration in hybrid Swainson’s thrushes, thereby linking ecologically relevant traits and fitness to better understand postzygotic isolation in this system.

## Methods

### 2.1. Data Collection

**Figure 1:**
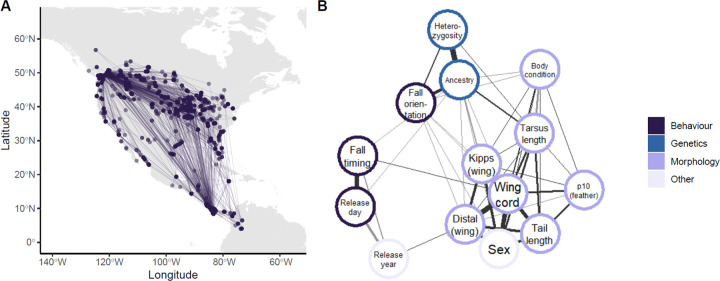
Phenotypic and survival dataset for juvenile Swainson’s thrushes. (A) Detections across the Motus Wildlife Tracking System for all birds. Points indicate the location of radio station detections, while lines were drawn between subsequent detections for the same individual. Points are drawn partially transparent, so darker points indicate that more birds were detected in the same location. (B) Phenotype network for all features included in the Random Forest model. Line thickness indicates strength of Spearman’s correlation estimated between features in the KNN-imputed dataset. Lines connecting uncorrelated features (based on significance from bootstrapping) not drawn.

We captured 479 juvenile Swainson’s thrushes using mist nets in the hybrid zone, near Pemberton, British Columbia, Canada. Juveniles were identified following Pyle (1997) and captures occurred during fall migration, from August to September in the years 2019 through 2023. Each bird was measured morphologically and blood samples taken for genetic analysis, following protocols approved by Institutional Animal Care and Use Committee at Texas A&M (IACUC 2019–0066) and under permits obtained from Environment and Climate Change Canada (SC-BC-2020–0016). To track birds on migration and quantify survival, we used the Motus Wildlife Tracking System—a network of automated radio telemetry stations that are found across North America [26] and includes our own fence of 14 radio stations. These 14 stations are spaced approximately 20 kilometers apart, and extend across the Swainson’s thrush hybrid zone from Delta to Kamloops in British Columbia, Canada. Each bird was fitted with a nanotag - a small radio transmitter that emits a unique signal detectable when birds fly within approximately 20 km of a Motus station - at capture. These birds pass by our fence of stations on their way south and other stations in the network before returning north to breed the next year (Fig. 1A) [20].

### 2.2. Phenotypic traits

Multiple morphological measurements were taken from each bird upon capture. Kipp’s distance, a measure of wing pointedness, was measured as the length between the longest primary feather and the first secondary feather (mm), while distal wing length was measured as the distance from p10 to the longest feather (mm). Additional measurements included tarsus length (mm), weight (g), tail length (mm), feather lengths (P7, P8, P9, P10; mm) and wing cord length (mm), which represent morphological traits expected to be part of the migratory phenotype [28, 29]. We calculated body condition as weight divided by tarsus length (g/mm), providing a proxy for overall energy stores. Three behavioral variables were analyzed: release day, fall migratory timing, and fall orientation. Release day was the day of the year a juvenile was tagged on the breeding grounds. Fall migratory timing was defined as the day of the year a bird was first detected by the Motus Wildlife Tracking System, within 30 km of its release site and during fall migration. Fall migratory orientation was calculated as the angle from the release site to the first Motus station where the bird was detected, within a 300 km radius.

To visualize relationships among phenotypic traits that were included in the Random Forest model (see Machine Learning Workflow) we built a phenotypic correlation network, following methods from Wilkins et al. (2015). Briefly, we estimated Spearman’s rank correlation between each pair of traits. We used bootstrapping with 100,000 replicates to determine which pairs of traits were significantly correlated with each other. We then plotted a network with lines drawn between all significantly correlated traits, with line thickness indicating the strength of the correlation (Fig. 1B).

### 2.3. Estimating survival

We used patterns of detections across the Motus Wildlife Tracking System as a proxy for survival. We considered detections over a 300 day study period, starting from the day each bird was tagged in the fall. We binned detections into 30 capture occasions of ten days, so that if a bird happened to pass by or stopover near a dense cluster of stations it did not cause a series of consecutive detections in the capture history [31]. If a bird was detected after the 300 day study period, it was marked as detected on the final capture occasion to reflect the fact that we know it survived. We ran a Cormack-Jolly-Seber model for each bird, to estimate its apparent survival probability given its capture history. We implemented this with the *crm* function from the R package *marked*, using the model “hmmCJS” and allowing both apparent survival and detection probability to vary across time [32]. To produce a survival probability across all capture occasions, we multiplied the survival probabilities estimated for each time point in the model. For all except one bird, apparent survival probability was either > 0.999 or < 0.001, allowing them to be clearly distinguished as apparently survived or apparently dead. The other bird had an apparent survival probability of 0.82 and was classified as ‘survived’.

### 2.4 Genomic ancestry

We used a standard phenol chloroform protocol to extract DNA and prepared libraries using methods adapted from Picelli et al. [33] and Schumer et al. [34] (see details in [20]). Genomes were sequenced to low coverage (median 3.55×) on an Illumina NovaSeq 6000. Following Justen et al. [20], we aligned sequences to both the inland and coastal reference genomes [35, 36], retaining reads that mapped to both references. We then called a panel of ancestry informative markers (AIMs), following a pipeline outlined in Blain et al. [37]. Briefly, we defined AIMs as markers with fixed differences between the inland and coastal reference genomes and with an allele frequency difference > 0.5 between a previously sequenced high coverage reference panel of 14 inland and 14 coastal birds [35]. We estimated read counts at each AIM, using a pipeline modified from ancestryInfer [38], then estimated ancestry at each autosomal AIM with ancestryHMM (parameters: -a 2 0.633 0.367 -p 0 −1000000 0.633 -p 1 −3000 0.367) [39]. We retained ancestry state calls with a posterior probability > 0.9. With plink, we filtered out AIMs with a minor allele frequency > 0.05 or missing data > 0.25, then performed linkage pruning (parameters: --indep-pairwise 200 20 0.2). This resulted in a final set of 1495 autosomal AIMs. With this AIMs set, we estimated ancestry as the proportion of inland versus coastal alleles, ranging from 0 for entirely coastal birds to 1 for entirely inland birds. We also estimated heterozygosity as the proportion of AIMs that were heterozygous, with values ranging from 0 for fully coastal or inland birds to 1 for F1 hybrids.

### 2.5. Machine Learning Workflow

To predict the binary outcome of migratory survival in Swainson’s thrushes, we developed a machine learning pipeline designed to optimize predictive accuracy, interpretability, and ecological relevance (Fig. S2). The response variable (phi_binary) encoded survival as 1 (detected in spring) and non-survival as 0 (not detected), based on automated telemetry data from the Motus Wildlife Tracking System. Predictor variables included 14 features related to structural morphology (distal wing length, p10, body condition, tarsus length, Kipp’s distance, tail length, wing chord), migratory behavior (fall_detectDay1, fall_bearing1, releaseDay, release_year), genetic ancestry (genome-wide ancestry and heterozygosity), and sex (sex_binary), as detailed in Section 2.2.

To reduce multicollinearity among features, we computed pairwise Pearson correlation coefficients and removed one variable from each pair with correlation ≥ 0.7 (Fig. S3a). This resulted in the exclusion of feather measurements P7, P8, and P9 due to their redundancy with wing chord, as well as the removal of body mass due to high correlation with body condition. Features with more than 25% missing data were excluded (Fig. S3b), with two exceptions—fall migratory timing and orientation—which were retained despite 28% missingness each, due to their strong ecological relevance. All features were standardized to have a mean of 0 and standard deviation of 1 (Fig. S4) prior to imputation, to ensure comparability across scales.

Missing data were imputed using the k-nearest neighbors (KNN) algorithm (Fig. S3C) [40], implemented via the KNNImputer function from scikit-learn [41]. KNN imputation estimates missing values by averaging the corresponding values of the k most similar samples (in this case, k = 5), identified using the nan_euclidean distance metric. We used uniform weighting for neighbor contributions and set a random seed (random_state = 42) to ensure reproducibility. Following imputation, the data were split into training and test sets using an 80%/20% stratified split, preserving the proportion of survivors and non-survivors in each subset. All preprocessing and modeling steps were conducted exclusively on the training data during model fitting to prevent data leakage.

The response variable exhibited substantial class imbalance, with 69.1% of individuals classified as non-survivors and 30.9% as survivors. To address this imbalance, we applied the Synthetic Minority Oversampling Technique (SMOTE) [42] to the training data using the imblearn package (v0.12.4) [43]. SMOTE generates synthetic minority-class samples by interpolating between randomly selected observations and their nearest neighbors, thereby preserving within-class variation while balancing the class distribution (Fig. S5).

For classification, we employed a Balanced Random Forest classifier [44], using the BalancedRandomForestClassifier implementation from imblearn.ensemble. This ensemble method balances class representation by under-sampling the majority class during bootstrap sampling, reducing bias toward the dominant class. The classifier was embedded in a scikit-learn pipeline that sequentially applied imputation, SMOTE, and classification. This structure ensured proper encapsulation of preprocessing steps and protected against information leakage during model evaluation.

Hyperparameter tuning was performed using randomized search with five-fold stratified cross-validation (RandomizedSearchCV), optimizing for the macro-averaged F1 score to balance precision and recall (two measures of model performance; see ‘Model Evaluation’) across classes. We tuned the number of trees (n_estimators: 100, 200, 300), maximum tree depth (max_depth: 10, 20, None), maximum number of features at each split (max_features: ‘sqrt’, ‘log2’, None), minimum samples required to split a node (min_samples_split: 2, 5, 10), and minimum samples required at a leaf node (min_samples_leaf: 1, 2, 4). Final model performance was assessed on the test set, and classification thresholds were adjusted to optimize recall and F1 scores for both survival and non-survival outcomes. This pipeline (Fig. S2) ensured methodological transparency and robustness while aligning predictive modeling with the biological realities of migratory survival.

### 2.6. Model Evaluation

A confusion matrix is a standard tool for evaluating classification model performance by comparing predicted outcomes with actual labels, such as survival status [45]. It comprises four categories: true positives (TP), true negatives (TN), false positives (FP), and false negatives (FN). In our case, true positives and true negatives indicate correct predictions of survival and non-survival, respectively. False positives represent instances where the model incorrectly predicts survival for individuals who did not survive, whereas false negatives occur when the model incorrectly predicts non-survival for individuals who actually survived. We used these categories to calculate key performance metrics, specifically: (1) accuracy (the proportion of all predictions that were correct), (2) precision (the proportion of predicted survivors that truly survived), (3) recall (sensitivity; the proportion of actual survivors correctly identified), (4) specificity (true negative rate; the proportion of actual non-survivors correctly identified), and (5) the F1 score (the harmonic mean of precision and recall, balancing both metrics to provide a single measure of positive class accuracy).

Model performance was assessed on the test set using accuracy, recall, and F1 score. We calculated these metrics separately for each class (i.e. birds that did and did not survive). Additionally, we tuned the classification threshold by varying it from 0.40 to 0.70 in 100 evenly spaced increments. For each threshold, confusion matrices and corresponding classification metrics were computed, with the optimal threshold selected as the one maximizing the macro-averaged F1 score (optimal threshold = 0.639).

To further evaluate the reliability of the model’s predicted survival probabilities, we computed the Brier score, a proper scoring rule that measures the mean squared difference between predicted probabilities and observed binary outcomes [46]. The Brier score ranges from 0 to 1, with lower values indicating better calibration and more accurate probabilistic predictions. A perfectly calibrated model achieves a score of 0, while a value of 1 reflects maximal disagreement between predictions and outcomes. Because probability calibration is particularly important for ecological interpretation, the Brier score was used alongside accuracy-based metrics to ensure that both the classification performance and probabilistic reliability of the model were adequately assessed.

Feature importance scores were derived from the model’s internal Gini importance metric, accessed via the .feature_importances_ attribute in scikit-learn (v1.2.2) [41]. This metric—also referred to as mean decrease in impurity—quantifies the total reduction in node impurity (Gini index) contributed by each feature across all trees in the forest. Features that result in larger reductions in impurity are considered more informative in partitioning the data. To contextualize the results, we compared each feature’s importance score against a null expectation (1 / number of features ≈ 0.07), representing the baseline importance if all features contributed equally.

To assess feature interactions in the Random Forest model, we conducted a permutation-based interaction importance analysis on the test set [47]. We first computed the baseline macro F1 score for the real test set using scikit-learn’s f1_score function. For each of the 91 unique pairwise combinations of features, we jointly permuted the values of both features across all samples in the test set, leaving the remaining features unchanged. This procedure disrupted any interaction effects between the two features while preserving their marginal distributions. We then measured the drop in macro F1 score relative to the baseline. To identify interactions with a meaningful impact on predictive performance, we retained feature pairs that caused a drop of ≥ 0.1 (i.e. 10%) in the F1 score.

To further interpret the model, we used SHAP (Shapley Additive Explanations) values calculated with the shap.TreeExplainer function from the SHAP Python package (v0.44.1) [48]. SHAP values quantify the contribution of each feature to individual predictions, providing detailed insight into how specific traits influence survival predictions. Values near zero indicate minimal influence, while larger absolute values reflect stronger effects. Monotonic trends in SHAP dependence plots suggest consistent feature effects, whereas scattered patterns imply more complex, possibly interaction-driven influences.

## Results

**Figure 2:**
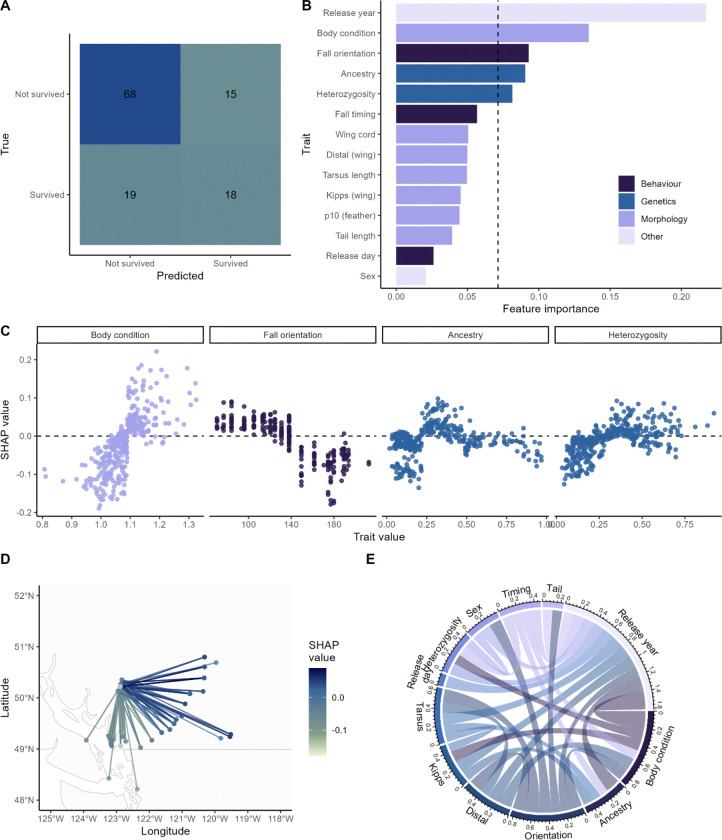
Random forest model evaluation. (A) Confusion matrix. The matrix shows counts of birds in the test dataset that were predicted by the model to have survived or not survived by their real survival status, with 68 true negatives, 15 false positives, 19 false negatives, and 18 true positives. (B) Feature importance plot. Feature importances were derived from the model using the mean decrease in impurity (Gini index). The dashed line indicates the threshold above which features are considered to have relatively high importance (1/number of features). (C) SHAP one-dimensional plots for top features. SHAP dependence plots illustrate how individual features influence the model’s output by showing the relationship between feature values and their contribution to predictions, with higher values indicating that they increase prediction of survival and lower values meaning that they increase prediction of non-survival. Each dot represents one bird from the training dataset, with the X-axis indicating the feature’s value and the Y-axis showing its SHAP value (contribution to the prediction). (D) Fall orientation estimates. Lines connect capture locations (more northern points) and locations where the birds were first detected within 300 km of the release site. Colour indicates the SHAP values associated with each measured orientation (positive values reflect higher predicted survival, negative values reflect lower predicted survival). (E) Interaction plot. Lines are drawn between features with a ΔF1 score > 0.1 (i.e. a 10% or more improvement in F1 score). Colours differentiate features associated with each line. Line thickness and the numbers in the legend indicate ΔF1 associated with each interaction.

The Random Forest model effectively predicted survival during migration, identifying a subset of traits with meaningful contribution, with an overall accuracy of 72%. We further validated model performance for each class (survival and non-survival) separately, which showed that prediction accuracy was primarily driven by effective prediction of which birds did not survive migration. For the non-survival class (class 0); precision was 0.78, recall was 0.82, and F1 score was 0.80, indicating that non-survival was predicted at a substantially higher rate than expecations under random chance. For the survival class (class 1), the model achieved a precision of 0.55, recall of 0.49, and F1 score of 0.51. While survival was not predicted better than random, these values do indicate that the effective prediction of non-survival was not driven by over-classification of the non-survival class.The confusion matrix showed 68 true negatives, 15 false positives, 19 false negatives, and 18 true positives (Fig. 2A).

Beyond classification accuracy, the Random Forest model demonstrated reliable probabilistic performance, as indicated by a Brier score of 0.235 [49]. This value suggests that predicted survival probabilities closely reflected observed outcomes, indicating that the model’s probability estimates were well-calibrated for ecological interpretation. In practical terms, individuals assigned higher survival probabilities by the model were more likely to have survived migration, supporting the model’s reliability in providing biologically meaningful probability estimates rather than merely categorical predictions.

Feature importance scores revealed that several variables had predictive effects that exceeded the null expectation, including release year, body condition, fall orientation, genetic ancestry, and heterozygosity (Fig. 2B). Permutation-based interaction importance analysis also revealed several feature pairs substantially contributed to model performance (Fig. 2E; Fig S6). The most influential interaction was between body condition and release year, which, when jointly permuted, reduced the macro F1 score by ΔF1 = 0.2275, suggesting a strong synergistic effect between individual condition and year-specific factors. Other high-impact interactions included ancestry with release year (ΔF1 = 0.1696), fall orientation with tarsus length (ΔF1 = 0.1644), and fall orientation with distal (ΔF1 = 0.1532). Notably, many of the top interactions involved release year, underscoring its role in modifying the effect of other predictors. Morphological variables (e.g., tarsus length, kipps, and distal) frequently appeared in important pairs, especially in combination with condition-related or temporal variables, suggesting that combinations of structural and physiological traits are critical for survival prediction. These results emphasize that interactions between biologically meaningful variables, rather than individual effects alone, can be central to understanding migratory outcomes.

SHAP dependence plots provided further interpretive insights into these variables (Fig. 2C). Birds with low to intermediate ancestry values (0.1–0.3), consistent with a more inland genetic background, were associated with higher predicted survival. Fall migratory orientation exhibited a negative relationship with probability of being classified as ‘survived’, associating more westward migratory directions with increased mortality risk (Fig. 2C–D). Higher body condition scores correlated positively with predicted survival. Heterozygosity values above 0.3 were linked to elevated probability of classification as ‘survived’. And lastly, individuals released in 2022 and 2023 were more likely to be predicted as survivors compared to those from earlier cohorts (2019, 2021).

## Discussion

Previous work in the Swainson’s thrush suggested migration serves as an extrinsic postzygotic barrier to gene flow in the system, with direct tracking data showing parental forms take different routes [16], hybrids exhibiting high variability and diverse combinations of migratory traits [19,20], and mark recapture models indicating at least some hybrid classes (early generation hybrids and coastal backcrosses) survive migration at lower rates than parental forms [21]. We used a Random Forest model to extend our knowledge of this system, leveraging this algorithm’s ability to model complex, nonlinear relationships and an autonomous system for tracking large numbers of songbirds on migration to identify key traits and interactions that shape survival on migration.

Our model achieved an overall accuracy of 72%, with precision and recall metrics indicating strong performance in predicting which birds would not survive migration. These validation metrics indicate that the model was effective at identifying individuals with combinations of phenotypes that reduce survival on seasonal migration. Greater predictability of non-survival, relative to survival, might not be unexpected. First, even adult birds breeding towards the middle of their species range (i.e., not in a hybrid zone) can have relatively low survival on migration due to the inherent risks associated with long distance travel as a small songbird [50]. Second, it is possible that novel trait combinations in hybrids will tend to act as incompatibilities, predictably reducing fitness in those birds [9], but well performing trait combinations will not increase survival to levels that would confer predictability in a classification model. While predictive performance of survivors was near chance (correct just over half the time) we still think this constitutes moderate predictive power given survival is a biologically complex outcome. Moreover, the relatively low Brier score (0.235) further supports that the model’s predicted survival probabilities were well-calibrated, providing confidence that its probabilistic outputs reflect meaningful biological variation in migratory risk.

Feature importance analyses revealed that genetic ancestry, heterozygosity, fall migratory orientation, body condition, and release year were consistently influential predictors of survival. Many of these traits showed evidence for interactions with other traits and SHAP dependence plots demonstrated that the relationships between traits and survival were not strictly linear or U-shaped. Combined, these results underscore the multifactorial nature of migration [28] and suggest that the expected pattern of disruptive selection may not fully explain survival outcomes in this system [51].

Variation in release year is likely related to changes in the extent of the Motus Wildlife Tracking System over time, while the observed positive relationship to body condition matches expectations that higher weight, relative to body size, should increase survival in migratory songbirds [52,53]. The observed variation in ancestry (higher survival of birds with low [inland] to intermediate ancestry), and to a lesser extent heterozygosity (higher survival at higher levels of heterozygosity), broadly matches prior results in the Swainson’s thrush. Blain et al. [21] used a subset of the present dataset to test if ancestry and/or heterozygosity predicted survival. Inland backcrosses (i.e., birds with mostly inland ancestry and elevated heterozygosity) exhibited some of the highest rates of survival in the system; early generation hybrids and coastal backcrosses (i.e., birds with mostly coastal ancestry and elevated heterozygosity) exhibited some of the lowest rates of survival. The generally higher survival of birds with more heterozygosity observed here contrasts to some extent with the observation that early generation hybrids had lower survival in the previous analysis but could be driven primarily by elevated survival of the inland backcrosses.

Migratory orientation, the direction of travel at the start of fall migration, had a feature importance that slightly exceeded that of ancestry and heterozygosity. This contrasts with prior attempts to link migratory behaviour to survival in this system [21], and suggests that effects of migratory behaviour may emerge only when considered in combination with body condition and genetic variation. Further, an assessment of pairwise interaction importance indicated that effects of migratory orientation may depend on a set of morphological traits, including tarsus, tail, and distal (wing) lengths. In our Random Forest model, birds with more eastward orientations tended to show higher survival. Because inland birds take more eastward migratory routes [16,54], this result aligns with the prior finding that hybrids with more inland ancestry tend to survive at higher rates than those with more coastal ancestry. However, this model extends our former work by implicating migratory behaviour associated with more inland genotypes in their survival.

For migratory divides to act as an extrinsic reproductive barrier, migratory traits are predicted to be tied to fitness. The traditional formulation of this hypothesis predicts that birds with intermediate migratory orientations should have low fitness [18]. Our results do not perfectly align with this hypothesis, but the low survival of more westward relative to more eastward orientations still suggests that the direction of migration ultimately impacts migratory survival, but with negative effects for some hybrid classes and positive effects for others. Stated another way, recombination in hybrids may be producing hybrid classes that occupy different locations on the fitness landscape. Lower survival in birds with a higher proportion of coastal ancestry and westward fall orientations may still help maintain reproductive isolation in the system but hybridization and migration may also be playing a creative role in the system, as birds with mostly inland ancestry, with elevated heterozygosity, and with more eastward fall orientations actually exhibit higher fitness than other hybrid/behavioral classes.

While Random Forest models offer robustness for ecological prediction, our approach has limitations. Machine learning approaches differ from traditional ecological models, which tend to be based on conventional statistical approaches, in a few key ways. Covariates (here, release year and sex) are not directly accounted for in classification as they would be for a statistical model. The form of the relationship between predictor and response variables is neither specified a priori nor returned in the model output – it is necessary to use downstream approaches (ex. SHAP) to understand how predictors influence the model outcome. While this can offer power in achieving model predictability in complicated ecological systems, it reduces model interpretability [55]. Furthermore, applying a classification model to this question required estimating survival as a binary response variable, which sacrificed our ability to jointly model survival and detection probability as in the capture mark recapture models that have previously been applied to measuring survival with Motus data [31,56]. Although our approach to estimating binary survival estimates takes individuals’ full capture histories into account, associations between capture frequency and specific traits were not accounted for here. However, we do not expect tower distributions to account for the low detection and inferred survival of birds with more coastal ancestry and more westward orientations, as our Motus station transect combined with additional stations maintained on Vancouver Island and northwestern Washington comprehensively spans the relatively narrow range of coastal subspecies routes into the breeding range [16,20]. Additionally, correlated selection among traits—a phenomenon well documented in evolutionary biology —was not explicitly accounted for here, potentially affecting our interpretation of variable importance [57]. Future studies could apply multivariate selection analyses to address this complexity. Finally, as with any associative model, caution is warranted in attributing causality to these predictors [58]. Nonetheless, machine learning models offer a powerful approach to disentangling relationships among ecological variables.

Future work could explore alternative machine learning frameworks, such as gradient boosting or neural networks, and incorporate a broader set of ecological covariates to enhance prediction accuracy and biological inference. Furthermore, environmental variables such as weather patterns and habitat quality, which can influence survival in migrating songbirds [31,59,60], were not included and could improve predictability in future modeling efforts [61]. Despite these limitations, our findings highlight the value of integrating genetic and phenotypic data through machine learning to deepen understanding of migratory survival and inform conservation strategies for migratory songbirds like the Swainson’s thrushes.

## Supplementary Material

Supplementary Files

This is a list of supplementary files associated with this preprint. Click to download.

• SupplementaryInformation.docx

## Data Availability

All sequences have been uploaded to the NCBI Sequence Read Archive under BioProject numbers PRJNA979932 and PRJNA1024534. Motus Wildlife Tracking System detections and all traits used in modelling have been uploaded to Dryad and a version of the repository that is accessible for peer review is available here: datadryad.org/share/LOMfdJYp63V_zDDCWv8jeOmfoW-UhkD_j8XLl3y-ZLU. The bioinformatics pipeline for calling ancestry informative markers is publicly available here: github.com/stephblain/thrush_ancestry_repeatability/tree/main/1_call_AIMs. All scripts for processing and modelling data related to this study are publicly available here: github.com/sarahvastani/swth_survival_ml.
